# A pain in the ACC

**DOI:** 10.1186/1744-8069-1-14

**Published:** 2005-03-31

**Authors:** Paul W Frankland, Cátia M Teixeira

**Affiliations:** 1 Programs in Integrative Biology and Brain & Behaviour, Hospital for Sick Children, Toronto, Canada; 2 Department of Physiology and Institute of Medical Science, University of Toronto, Toronto, Canada

## Abstract

An emerging theme in systems neurobiology is that even simple forms of memory depend on activity in a broad network of cortical and subcortical brain regions. One key challenge is to understand how different components of these complex networks contribute to memory. In a new study in *Molecular Pain*, Tang and colleagues use a novel set of approaches to characterize the role of the anterior cingulate cortex (ACC) in the formation of Pavlovian fear memories.

Because survival may depend upon it, we learn about painful stimuli quickly and efficiently through a process known as Pavolvian fear conditioning [1]. Viewed in this framework, stimuli present in the environment at the time of injury (*or conditioned stimuli*; CSs) are likely to become associated with the painful stimulus (*or unconditioned stimulus*; US). This is adaptive since when these cues are next encountered, an animal can take measures (*or conditioned responses*; CRs) that help to reduce the likelihood of injury.

A painful stimulus may have a number of different attributes [2]. These include sensory-discriminative attributes, such its location, intensity and quality, as well as affective attributes, such as its *unpleasantness *(or negative emotional valence). Recent studies in humans and experimental animals have established that these different attributes are processed by partially dissociable brain networks [2,3]. For example, in human imaging studies activity in the primary somatosensory cortex is closely related to the sensory-discriminative properties of a painful stimulus, whereas activity in the anterior cingulate cortex (ACC) is closely related to the affective features of pain, such as subjective feelings of unpleasantness [4]. Taking their lead from these studies, Tang and colleagues [5] first asked whether the ACC plays a similar role in processing the affective features of pain stimuli in mice. They reasoned that activation of the ACC should evoke a similar set of behaviors as would presentation of an actual painful stimulus, such as a footshock. Consistent with this, they first show that electrical stimulation of the ACC evokes high frequency ultrasonic vocalizations – a typical sign of distress in rodents. Similarly, chemical stimulation of these neurons evokes immobility or freezing – species-typical signs of fear. While it is difficult to evaluate subjective feelings of unpleasantness in animals, this set of behaviors evoked by ACC activation is clearly consistent with such an affective state.

Having established that activation of the ACC produces a cluster of behaviors consistent with fear or unpleasantness, Tang and colleagues posed a more ambitious question: Would it be possible to artificially fear condition an animal by replacing a footshock with electrical stimulation of the ACC? In a normal fear conditioning experiment, an otherwise neutral stimulus, such as a tone, is repeatedly paired with an aversive stimulus, such as a shock, in a particular context. Later an animal exhibits a number of species-typical fear responses, including freezing behavior, when presented with the tone or placed back in the original training context. Here, Tang and colleagues conducted the same experiment except that they replaced the footshock with stimulation of the ACC. Sure enough, mice trained in this way froze when placed the mice back in the same context, or when presented with the tone in a different context. These artificially-generated conditioned fear memories were not transient – they lasted at least 3 days, indicating that mice will condition as readily to ACC stimulation as they might to shock delivery. This experiment, therefore, shows that mice readily associate both the context and the tone with ACC-evoked 'unpleasantness'. The formation of CS-US associations underlying fear memories depends on NMDA-mediated plasticity in the amygdala [6-8]. Consistent with this, Tang and colleagues went on to show that the formation ACC-induced fear memories is disrupted by localized pharmacological blockade of NMDA receptor function in the amygdala. This series of experiments demonstrates, therefore, that an ACC-induced fear memory looks and behaves very much like a fear memory induced by footshock (Figure 1).

**Figure 1:**
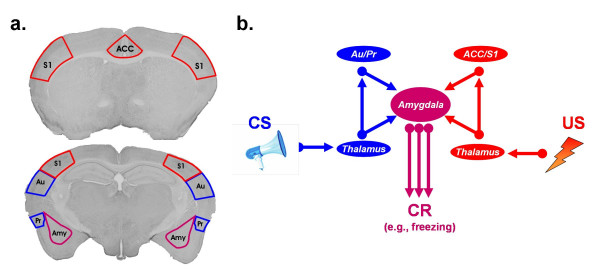
The anatomy of Pavlovian fear. **a**. Mouse brain sections illustrating key brain regions involved in processing CS information (blue), US information (red) and CS-US associations (purple). **b**. Simplified schematic of pathways involved in the formation of Pavolvian fear memories. Direct and indirect connections from thalamic nuclei convey information about the CS and US to the amygdala. Tang and colleagues provide evidence that the ACC processes the affective features of painful stimuli, and that pairing a tone with ACC activation can artificially induce Pavlovian fear memories. Abbreviations: ACC, anterior cingulate cortex; Au, primary auditory cortex; Amy, amygdala; Pr, perirhinal cortex; S1, primary somatosensory cortex.

Tang and colleagues took care to rule out a number of alternative interpretations. They first showed that the fear conditioning to the tone depended on the *pairing *of the tone with ACC stimulation. When this temporal relationship was perturbed, the mice did not freeze to the tone, indicating that the observed learning was associative in nature. A second issue they addressed was the possibility that ACC stimulation produces analgesic effects [9]. If this were the case, then ACC stimulation would be expected to inhibit, rather than facilitate, aversively-motivated learning as well as behavioral responses to noxious stimuli. Here, Tang and colleagues showed that ACC stimulation potentiates responses in the tail-flick and hot-plate tests, and facilitates learning in a hot-plate avoidance task [10]. These data demonstrate that ACC activation, rather than producing analgesia, facilitates responses to noxious stimuli in a broad range of experimental situations.

A third control experiment yielded a particularly interesting dissociation. They considered the possibility that brain stimulation, regardless of site, is aversive. To address this they conducted the same experiment but paired a tone with stimulation of the primary somatosensory cortex. Under these conditions, no fear developed to the tone (nor to the context in which conditioning occurred). This first demonstrates that their effects are specific to ACC stimulation, and secondly, suggests an important dissociation. Whereas the sensory features of a pain stimulus are processed by the primary somatosensory cortex and the affective features by the ACC, only ACC stimulation appears to functionally substitute for a shock during fear conditioning. This suggests that fear conditioning critically depends on the affective qualities (or emotional valence) of the US. When the US is stripped down to its sensory properties alone, conditioning proceeds very slowly (or not at all). This conclusion is further supported in another experiment in which mice were fear conditioned with a tone-shock pairing. Tang and colleagues showed that inactivating the ACC during training blocked the development of conditioned fear. This suggests that disrupting processing of the affective (but not sensory) features of a US is sufficient to disrupt conditioning.

This clever series of studies starts to dissociate the contribution of different cortical regions to the formation of even simple memories. Tang and colleagues provide evidence that the ACC specifically processes the affective features of painful stimuli, and that ACC activation can artificially induce Pavlovian fear memories. But how do these findings fit into the broader context of functional studies on the ACC? While other rodent studies have also provided evidence that the ACC processes Pavolvian fear memories, these have emphasized the role of the ACC in memory recall rather than memory formation [11,12]. Furthermore, these studies provide evidence that the ACC is preferentially involved in the recall of remote (rather than recent) fear memories, and this role in processing remote memories extends to appetitively-motivated spatial tasks [13,14]. When we also consider functional imaging studies in human subjects showing that, besides processing pain stimuli, the ACC is involved in a large number of different cognitive processes (e.g., attention, error monitoring, target detection and effortful recall) [15], it becomes clear that we are a long way from a complete understanding of the ACC [16]. In some instances it is possible that the ACC performs similar functions across situations – for example, retrieval of remote, rather than recent, memory may involve more effortful recall. However, the diversity of function attributed to the ACC suggests that this region is functionally heterogeneous, and a single model is unlikely account for all observations.
